# Exploring Scottish addiction services: provider-based stigma, addiction aetiology beliefs, treatment bias, and burnout among addiction treatment providers

**DOI:** 10.1186/s13011-026-00701-0

**Published:** 2026-01-22

**Authors:** Beata Ciesluk, Greig Inglis, Adrian Parke, Lucy J. Troup

**Affiliations:** 1 School of Education and Social Sciences, University of the West of Scotland, Paisley, Scotland, UK; 2 Norwich University of the Arts, Norwich, United Kingdom of Great Britain and Northern Ireland

**Keywords:** Addiction treatment providers, Provider-based stigma, Burnout, Harm reduction, Abstinence, Addiction aetiology

## Abstract

**Background:**

Drug related deaths continue to increase in Scotland. Many barriers to addiction treatment exist and are often related to poor provider-client relationships possibly caused by stigma, burnout and differentiating beliefs and attitudes among addiction treatment providers. This study investigated the prevalence of provider-based stigma (PBS) including four stigma variants (dangerousness, blame, social distance, fatalism) and its relationship to burnout, job satisfaction, attitudes towards addiction treatment approaches, and beliefs regarding addiction aetiology in a sample of addiction treatment providers.

**Methods:**

Cross-sectional online survey was completed by 64 addiction treatment providers currently working in Scotland. Online survey was comprised of validated and adapted measures, extensive statistical analysis was conducted, including ANOVAs and Regressions to examine the outcomes of interest.

**Results:**

Over 30% of participants had elevated scores on PBS variants dangerousness and blame and these were found to predict higher acceptance of abstinence-based treatments, and lower endorsement of harm reduction approaches (dangerousness: *b* = 0.41, *p* < 0.001; blame: *b* = 0.23, *p* = 0.010). Burnout was high in this sample, and PBS variant blame predicted higher client-related burnout (*b* = 7.35, *p* = 0.009). Moreover, the belief in the disease model predicted higher acceptance of abstinence-based treatments (*b* = 0.30, *p* < 0.001), whereas belief in the psychosocial model predicted higher acceptance of harm reduction-based treatments (*b* = -0.25, *p* = 0.008). Lastly belief in the disease model predicted higher scores on dangerousness (*b* = 0.19, *p* = 0.016) and fatalism (*b* = 0.29, *p* = 0.002) PBS variants.

**Conclusions:**

The findings from this study provide insights for policy and addiction treatment improvements. Efforts to alleviate addiction treatment providers stigmatising attitudes, especially perceptions of people who use drugs as dangerous, blameworthy, and incapable of recovery are needed. Interventions and policy improvements need to include stigma reduction workshops and consider addiction treatment providers attitudes and beliefs to limit treatment bias, stigma and burnout to foster better relationships between clients and the workers who support them.

## Introduction

In 2023, drug related deaths in Scotland reached an average of 277 drug-related deaths per million population, which exceeded the rates of many European countries including other UK nations [1]. This decade long public health crisis has caused extensive individual, societal and economic burden [2]. The Scottish Government has recently highlighted a need for improvement in addiction services to combat this crisis. In Scotland, addiction treatment provision is complex and varied, mainly funded by public health and statutory services but also often provisioned by third-sector organisations. Services offered include outreach support, abstinence-based support, as well as recently increasing provision of harm reduction practices, including medication assisted treatment (MAT), naloxone provision and opening of the first drug consumption rooms [3–5]. Due to this diversity, efficiency of addiction provision in Scotland is difficult to assess, and views of its effectiveness remain mixed. To account for this, and achieve overall service improvements, the Scottish Government has called for the creation of an appropriate workforce with the “right attitudes, training and supervision” [5] and an urgent need to reduce stigma towards people with substance use disorders (SUDs) recognising it as one of the most significant barriers to addiction treatment initiation, maintenance and recovery [5, 6]. Interviews with individuals using treatment services in Scotland confirm the need for change, reporting treatment dissatisfaction and high dropout rates due to poor relationships with addiction treatment providers caused by discrimination, paternalism and staff turnover disrupting continuity of care and development of therapeutic relationships [7, 8]. Therefore, understanding how we can improve the existing addiction workforce in Scotland is necessary.

One area that warrants further investigation which could inform and improve treatments is a type of stigma called Provider-Based Stigma (PBS), which comes directly from treatment providers, and is directed towards individuals receiving care [9]. PBS is prominent across most health conditions but especially in SUDs [10–12]. PBS has been associated with perceived lower quality of treatment and poor provider-client relationships [13, 14]. This correlates with findings that PBS also leads to lower job satisfaction [15, 16], increased turnover, and burnout among providers [17–19], with all these aspects found to directly impact the development of therapeutic relationships [17–19]. However, research on PBS towards individuals with SUDs is limited, as it is often conducted within the US, in medical or private treatment settings and is therefore not generalisable to Scottish addiction services which are usually publicly funded [19, 20]. Moreover, most of this research uses vague definitions of PBS which are difficult to measure [9].

Recently, researchers aimed to improve research into PBS towards individuals with SUDs with the development of a new measure which investigates four distinct PBS variants: perceptions of dangerousness (the belief that people with SUD threaten the safety of community), *blame* (belief that people with SUD are responsible for their condition), *social distance* (desire to avoid social interactions and discomfort living nearby individuals with SUD), and *fatalism* (fatalistic beliefs regarding SUD recovery) [21]. Subsequent research incorporating these variants found that they were all highly prominent across many service domains (e.g., criminal justice, healthcare, education and drug treatments) [22–24]. Furthermore, findings from these studies indicate that some PBS variants, specifically heightened perceptions that individuals with SUDs are dangerous and responsible for their conditions, were related to lower support for harm reduction treatments. In addition, fatalistic beliefs regarding SUDs recovery were associated with disapproval of methadone and naloxone provisions, and poor overdose prevention practices [21–24]. Notably, to date no studies have examined these variants of PBS in Scottish addiction services. Given their prominence, and influence on workforce quality and treatment delivery, the current study aims to understand PBS in Scottish addiction treatment providers, and its potential influence on burnout levels, job satisfaction, and attitudes towards addiction treatment approaches.

Another factor that could affect addiction treatment attendance, quality and delivery is treatment providers’ beliefs regarding addiction aetiology. These beliefs have been shown to influence the way people who use drugs are characterised, diagnosed and treated [25, 26]. Specifically, certain beliefs cause individuals to receive inconsistent treatment regimens which heightens treatment dissatisfaction and dropout rates [27]. There is also evidence to suggest that the belief in the disease model of addiction is associated with acceptance of different treatment approaches. Findings show that treatment providers who support the model are more likely to insist on abstinence-based treatments and are opposed to harm reduction interventions [27]. Conversely other studies found that the belief in the disease model has been positively associated with harm reduction-based treatments such as medication assisted treatment (MAT) and naloxone provisions [22]. Given the rising popularity and effectiveness of harm reduction approaches in Scotland [4, 28, 29] it is crucial to explore if providers’ beliefs may affect the acceptance of these interventions. Lastly, recent research indicates that certain beliefs may be associated with PBS, with some studies showing that the belief in the disease model can increase optimism regarding SUDs recovery and decrease perceptions of blame [21] whereas others show that it can increase perceptions that individuals with SUD are dangerous and unlikely to recover [30]. Overall, the research in this area is notably scarce, inconsistent, and conducted mainly in the US, but does demonstrate that beliefs in addiction aetiology could impact treatment delivery and PBS. The current study aims to investigate addiction aetiology beliefs and their influence on Scottish addiction services.

Therefore, this study aims to build on this existing research and investigate potential targets for treatment improvements by evaluating four variants of PBS, job burnout, job satisfaction, attitudes towards addiction treatment approaches and addiction aetiology beliefs in Scottish addiction treatment providers. By investigating these aspects of Scottish addiction workforce, we aim to provide an outline for future research which will be important for improving addiction policies and interventions in Scotland.

## Methods

### Design

A cross-sectional design was used to collect data online using Question Pro between April 2024-September 2024. The target sample was addiction treatment providers, over 18 years old, currently working with or supporting individuals in Scotland with current or past SUDs.

Participants were invited to complete the survey through social media posts, emails and newsletter invitations from government, third sector officials and addiction networks. Overall, there was 2515 survey views, and 200 individuals started the survey with a completion rate of 33.5% (*N* = 67). Three participants were excluded as they did not currently work in Scotland, leaving a total of 64 participants.

This study has been preregistered 10.17605/OSF.IO/WKHMN.

### Measures

The online survey was developed by a multi-disciplinary team of researchers, including a clinical cognitive neuroscientist, and a person with drug and alcohol work experience. The survey consisted of demographic questions (Table 1) and the following measures: Provider-Based Stigma questionnaire [21, 22] which assessed 4 stigma variants: *Dangerousness*,* Blame*,* Social Distance* and *Fatalism*, Copenhagen Burnout Inventory (CBI) [31], which assessed participants *personal burnout* (PB), *work-related burnout* (WB) and *client-related burnout* (CB), Harm Reduction Acceptability Scale (HRAS) [32] which assessed participants attitudes towards addiction treatments approaches specifically acceptance of *harm reduction-based treatments* and *abstinence-based treatments*, Short Understanding of Substance use Scale (SUSS) [33] which assessed participants belief regarding addiction aetiology, beliefs of the *psychosocial model* and *disease model*, and a one item measure, assessing participants job satisfaction [34]. Several questions were adapted to suit the context of the current study. Table 2 describes all the scales, and any adaptions made.

**Table 1:** Demographic characteristics of the sample (*N* = 64)

Baseline Characteristic	*N*	%
**Gender**		
Female	44	68.70
Male	19	29.60
Trans Woman	1	1.56
**Age Group**		
18–24	3	4.68
25–34	18	28.13
35–44	10	15.62
45–55	21	32.81
56–70	12	18.75
**Length of Service (years)**		
0–1	5	7.81
2–5	18	18.13
6–10	12	18.75
11–15	8	12.50
16–20	9	14.06
21+	12	18.75
**Role** ^**a**^		
Counsellor Therapist	7	10.94
Social Worker	1	1.56
Support Worker	15	23.44
Care Manager	7	10.94
Programme Manager	4	6.25
Peer Support Worker	3	4.60
Outreach Worker	4	6.25
Drug Service Worker	22	34.38
Other	17	26.56
**Work Description** ^**b**^		
Nonhospital	2	3.13
Hospital Base	2	3.13
Outpatient Clinic	17	26.56
Day programme	5	7.81
Residential Rehab Centre	10	15.60
Community Based Support Groups	15	23.43
Online Support Service	2	3.13
Other ^c^	21	32.81
**Treatment Type** ^**d**^		
Harm Reduction	57	89.06
Psychosocial – Motivational Interviewing (MI)	51	79.69
Psychosocial – Relapse Prevention	49	76.56
Psychosocial – Cognitive Behavioural Therapy (CBT)	35	54.69
Psychosocial – Family Work	23	35.94
Counselling	30	46.88
Pharmacological	33	51.56
Other ^f^	11	17.19

*Note. All answers are self-reported using the provided names of roles and treatment types*,* formal definitions of these were not provided to participants during survey completion to avoid response bias*

^a^ measured by *“Please choose which of the following best describes your role*,* select any that apply”*

^b^ measured by *“Which treatment setting best describes your current role*,* please select any that apply”*

^c^ Open answers included following themes: *prison based*,* homeless accommodation*,* nursing*,* recovery services*,* outreach*,* social work*

^d^ measured by “Which, if any of the following treatment types does your organization offers? Please select any that apply”

^f^ Open answers included following themes: *safe injection provisions*,* blood borne virus testing*,* sexual health testing and treatment*,* trauma support*

**Table 2:** Descriptions of scales used in the online survey

Measure	Description
Provider-Based Stigma (PBS) Questionnaire [21, 24]	This questionnaire is comprised of four measures of PBS including: *Dangerousness* (the degree to which participants believed drug users threaten the safety of their community), *Blame* (belief that drug users are responsible for their condition), *Social Distance* (desire to avoid social interactions and discomfort living nearby individuals with SUD), and *Fatalism* (fatalistic beliefs regarding SUD recovery). Higher scores on each measure indicate greater PBS. Each statement was rated on a 5-point Likert scale, ranging from “*Strongly Agree*” to “*Strongly Disagree*.” The reverse-coded responses were summed and averaged to create scores for each stigma variant. This questionnaire was adapted as the original questions focused on opioid-use disorder only (e.g. “If I knew that a heroin addict lived nearby, I would not allow my children to play alone outside.”), as the current study focus was all types of SUDs, we adapted words such as “heroin/opioids” to “drugs/addiction.” The *Dangerousness* (α = 0.76) and *Social Distance* (α = 0.76) measures had good internal reliability. *Blame* (α = 0.68) and *Fatalism* (α = 0.60) had lower internal consistency but were still considered reliable due to strong item loadings and good inter-item correlations (*r* > 0.2) **(21).** In our sample, Dangerous and Blame showed good internal consistency (α = 0.80 and α = 0.72) whereas Social Distance (0.68), and Fatalism had lower internal consistency (0.40) but were also still considered reliable due to good inter-item correlation (*r* > 0.2)
Copenhagen Burnout Inventory (CBI) [31]	A 19- items scale, consisting of three subscales: Personal Burnout (PB) (9 items), Work-related burnout (WB) (9 items) and Client-related burnout (CB) (6-items). Responses were measured on a 5-point Likert scale which changed depending on the wording of the item (Always, Often, Sometimes, Seldom, Never/Almost never, or to a very high degree, To a high degree, Somewhat, To a low degree, To a very low degree). Depending on the scale used the responses were measured as follows: 0 = *never*, to 100 = *always*, or 0 = *very rarely*, to 100 = *very often*. Only one item was reverse-coded. Responses were summed and averaged to create the score for each subscale. PB Subscale refers to the generic feeling of physical and psychological exhaustion experienced by the person, regardless of their occupation status (e.g., “How often do you feel tired?”, “How often do you feel worn out”), WB subscale refers to the degree of which physical and psychological fatigue and exhaustion is related to participants occupation (e.g., “Is your work emotionally exhausting?”, “Do you feel burnt out because of your work?”). CB subscale refers to the state of prolonged physical and psychological exhausted which is perceived as related to the work with clients/patients (e.g., “Are you tired of working with clients,” “Do you find it frustrating working with clients”). This scale has satisfactory level of reliability and validity (α 0.85–87) [31].
Harm Reduction Acceptability Scale (HRAS) [32]	A 25-item measure with a 5-point Likert Scale (1 = *strongly agree*, 5 = *strongly disagree*), with 12 items being reverse scored. Mean score above 3 indicates favourable attitudes towards *abstinence-based treatments* and mean scores below 3 indicates favourable attitudes towards *harm reduction-based treatments*. It has a high internal consistency and a moderate test-retest reliability with Cronbach alpha ranging from 0.89 to 0.93 *r* = 0.83 [32]
Short Understanding of Substance use Scale (SUSS) [33]	A 19-item scale measuring different beliefs regarding substance use, containing 3 subscales: *psychosocial model* (5-items), *disease model* (7-items) and *eclectic orientation* (7-items), with each item rated on a 5-point Likert scale (0 = *strongly disagree*, 4 = *strongly agree*). Consistent with previous work (Barnett et al., 2018, Moggi et al., 2005) due to lack of reliability *eclectic orientation* subscale was excluded from the current analysis. *Disease model* in this scale refers to the view that SUDs are chronic, progressive illnesses, curable only by lifelong abstinence and caused partially by genetic predisposition and neural changes due to prolonged substance use. Example of an item “There are only two possibilities for an alcoholic or drug addict – permanent abstinence or death”. *Psychosocial model* refers to the view that SUDs are learned and shaped by cultural, social and familiar backgrounds, and can be managed through various interventions such as harm reduction techniques, cognitive behavioural therapy and improvements in coping and social skills. Example of an item “A person can develop alcoholism or drug addiction because of underlying psychological problems.” Higher scores on each measure indicate higher endorsement of the model. SUSS has good internal consistencies with *r* = 0.78 for the Disease Model and *r* = 0.075 for the Psychosocial Model [33].
Job Satisfaction [34]	Job satisfaction was measured by a single item “How satisfied are you with your current job” on a 5-point Likert scale (1 = *completely dissatisfied*, 5 = *very satisfied*)

### Data analysis

Data was curated in Excel (Microsoft 365, Microsoft, WA, USA). After descriptive analysis of demographic information (Table 1) and primary outcomes (Table 3), we used ANOVAs, t-tests, and Pearson’s correlations to asses’ differences within continuous outcomes and chi-square tests within categorical variables. The predictive influence of PBS on burnout subscales scores (PB, WB, CB) and on attitudes toward treatment approaches i.e. HRAS scores was assessed via separate multiple linear regressions using a backward data entry. This method was used due to the limited sample size, exploratory nature of the study and lack of previous work including these scales in a regression model [35].

**Table 3:** Descriptive statistics (*N* = 64)

Variable	M	SD	Min	Max
**PBS**				
*Dangerousness*	1.78	0.66	1.00	3.30
*Blame*	1.82	0.77	1.00	4.00
*Social Distance*	1.77	0.60	1.00	3.20
*Fatalism*	1.62	0.79	1.00	4.00
**Burnout** ^**a**^				
PB	50.13	18.95	17.00	100.00
WB	41.96	19.54	14.00	93.00
CB	27.21	17.44	0.00	67.00
**HRAS**	1.84	0.60	1.00	3.70
**Job Satisfaction** ^**b**^	4.10	0.72	1.00	5.00
**Addiction Aetiology** ^**c**^				
*Disease Model*	1.34	1.05	0.00	4.00
*Psychosocial Model*	2.88	0.66	1.00	4.00

Note. ^a^ Measured by Copenhagen Burnout Inventory (CBI)

^b^Measured by Job Satisfaction item “How satisfied are you with your current job”

^c^Measured by Understanding Substance Use Scale (USUS)

HRAS = Harm Reduction Acceptability Scale, PBS = Provider-Based Stigma, CBI = Copenhagen Burnout Inventory, PB = Personal Burnout, WB = Work-Related Burnout, CB = Client-Related Burnout

The relationship between PBS and job satisfaction was assessed via Spearman’s rho, as normal distribution tests illustrated deviation from normality on the job satisfaction item (Shapiro wilk test: 0.75, *p* < 0.001; Kurtosis: 4.50; Skewness: -1.23).

Differences in addiction aetiology beliefs were assessed by repeated measures ANOVA. Relationship between those beliefs and attitudes towards addiction treatment approaches were assessed by Pearsons’s correlations, and multiple linear regression. Lastly, to establish the predictive relationship of these beliefs on PBS, four separate multiple regression models were performed on each PBS variant. All data was analysed using JASP Team (2022). JASP (Version 0.16.3) Microsoft Windows 10)

## Results

### Demographics

Table 1 displays the characteristics of the participants.

The sample was comprised of mostly female participants (68.70%). Race or ethnicity was not collected for this sample. To take part, all participants were required to work in Scotland, therefore it is likely the sample is relatively homogeneous. There were a variety of roles in the sample, including Drug Service Workers (34.38%), Counsellor Therapists (10.94%), Support Workers (10.94%), and Management positions (17.10%). Most participants reported working in Outpatient Clinics (26.56%), Community Based Support (23.43%) and Residential Rehab Centres (15.6%). Main treatment types provided included Harm Reduction (89.06%), Psychosocial – MI, (78.69%), Relapse Prevention (76.56%) and Cognitive Behavioural Therapy (CBT) (54.69%).

### Age and length of service

To assess if age or length of service was associated with any outcomes of interest, Pearsons’s correlations were conducted. Results indicated that only age had a significant correlation and was negatively correlated to CB (*r* = -0.27, *p* = 0.003). As this was a weak correlation and no other correlations were found to be significant, age and length of service were not included in any subsequent analysis.

### Comparisons and distribution of PBS variants

Descriptive statistics showed that participants’ scores on the four PBS variants (*Dangerousness*,* Blame*,* Social Distance*,* Fatalism*) were low (*M* < 2.00) with some subscales displaying higher means (e.g. Blame) than others (e.g. Fatalism) (Table 3). Repeated measures ANOVA did not identify statistically significant differences between these means (*p* > 0.05) however each PBS variant was retained for its potential predictive effect on burnout, attitudes towards addiction treatment approaches, job satisfaction, and its relationship to addiction aetiology beliefs.

**Fig. 1:**
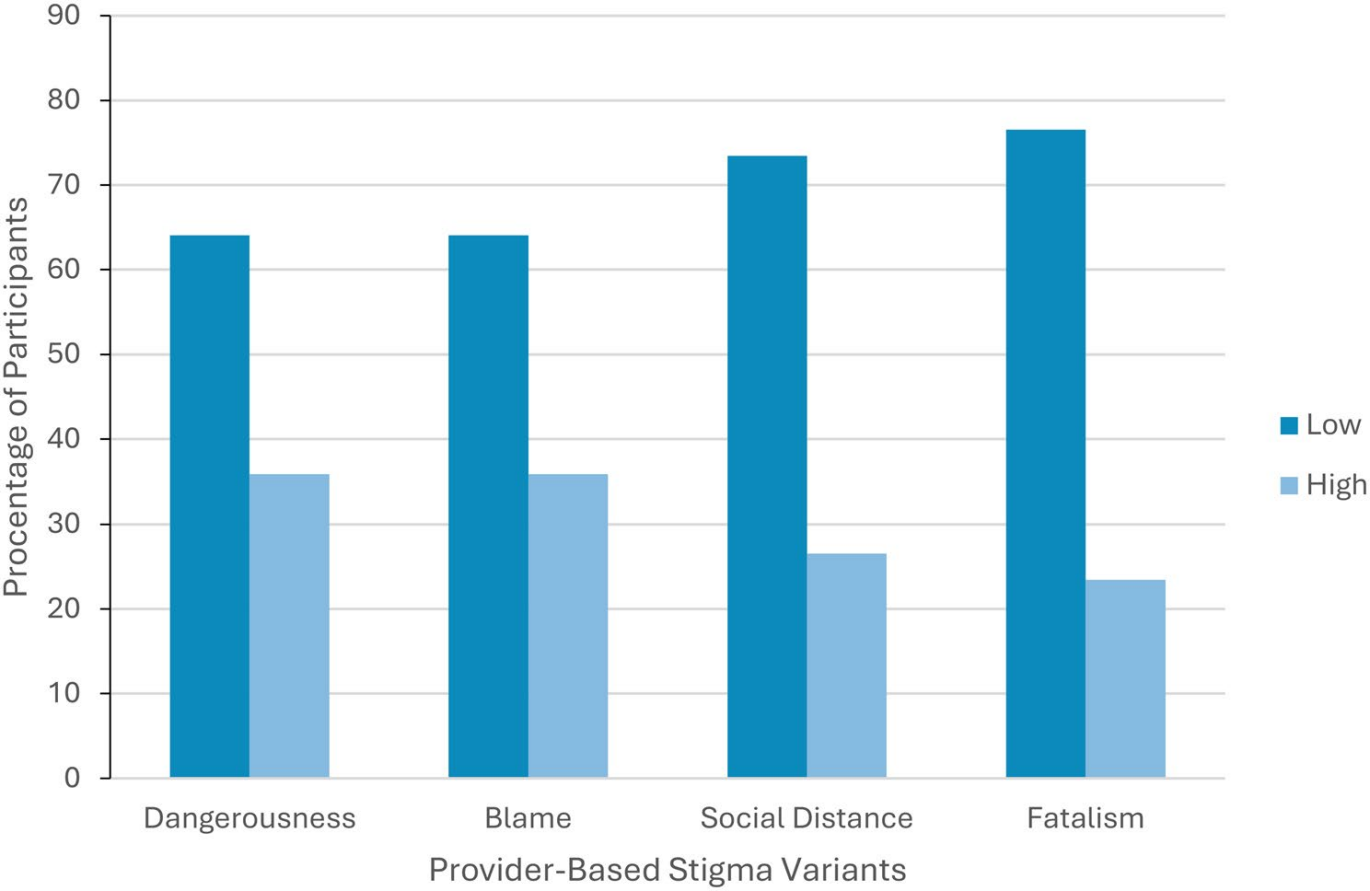
Distribution of participants across the four stigma variants

To further investigate PBS prevalence, participants were assigned a low or a high level of PBS based on their average Likert point score (0–4) on each variant. This was conducted by assigning low level of PBS to those scoring below the midpoint (< 2) and high level to those scoring above midpoint (> 2) [21, 22]. Proportions for each level were then converted into percentages. As seen on Fig. 1, certain PBS variants were prominent, with over 30% of the participants having high scores on *Dangerousness* and *Blame* variants.

### Comparisons and distribution of burnout subscales

A repeated measures ANOVA using the Huynh-Feldt correction was conducted to examine the differences in means across the three burnout subscales: PB, WB, CB.

PB and WB mean scores were high, and CB were comparably low in this sample (Table 3). Significant differences between these means were found *F*(1.63, 102) = 55.58, *p* < 0.001 *η²* = 0.47 with Post Hoc analysis showing that PB was significantly higher than WB and CB ( *p* <. 001). WB was also significantly higher than CB (*p* < 0.001*)* (Table 4).

**Table 4:** One-Way repeated measure ANOVA using Huynh-Feldt correction for burnout subscales (*Personal burnout*, *Work-Related burnout*, *Client-Related burnout*) with Post-hoc comparisons using Holm-Bonferroni correction

Measure	SS	df	MS	F	*p*	η²	Post Hoc Test			
							MD	95% CI LL UL	t	*p*
**Burnout** ^**a**^	17267.68	1.63 102.76	10586.11	55.58	< 0.001	0.47				
**Error**	46285.13	63	734.69							
PB vs. WB							8.17	2.82 13.51	3.70	< 0.001
PB vs. CB							22.92	17.57 28.26	10.40	< 0.001
WB vs. CB							14.75	9.40 20.09	6.70	< 0.001

Note. ^a^Measured by Copenhagen Burnout Inventory

*SS* = sum of squares, *MS* = mean square, *MD* = mean difference, 95% CI LL, UL = lower and upper limits of a confidence interval for *MD*, PB = *Personal Burnout*, WB = *Work-Related Burnout*, CB = *Client-Related Burnout*

To further analyse the distribution of participants’ scores on the burnout subscales the scores for each subscale were categorised into low (*M* ≤ 25), intermediate (*M* > 25 - ≤ 49) and high levels (*M* ≥ 50) of burnout based on participants’ mean scores. Proportions for each level were then converted into percentages.

**Fig. 2:**
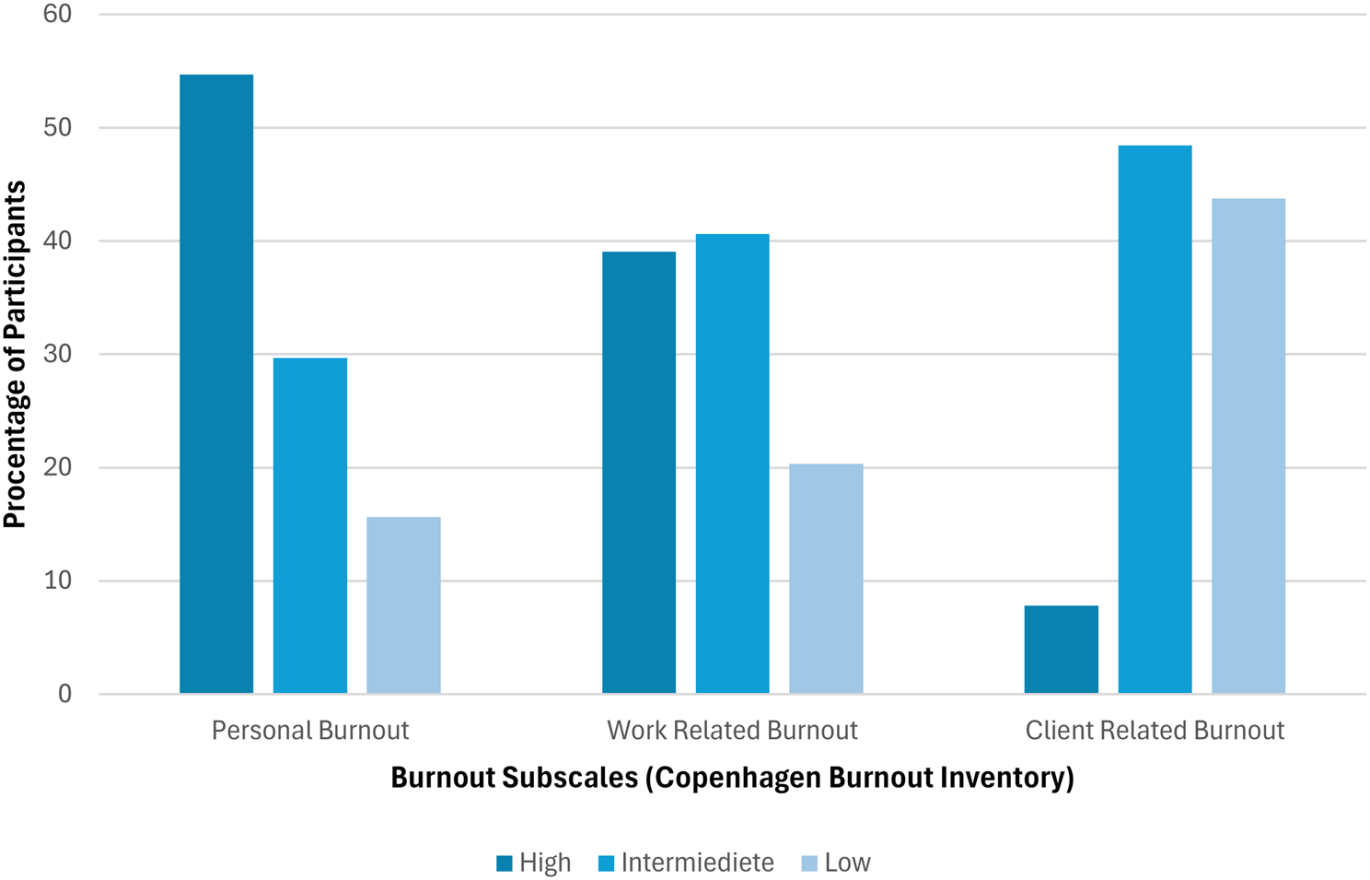
Distribution of participants across three burnout subscales

As seen on Fig. 2, PB had the highest proportion of participants in the “high” level of burnout, CB has the highest proportion of participants in “low” burnout levels, and CB and WB had similar proportions of participants in the “intermediate” levels of burnout.

### Relationship between PBS and burnout

**Fig. 3:**
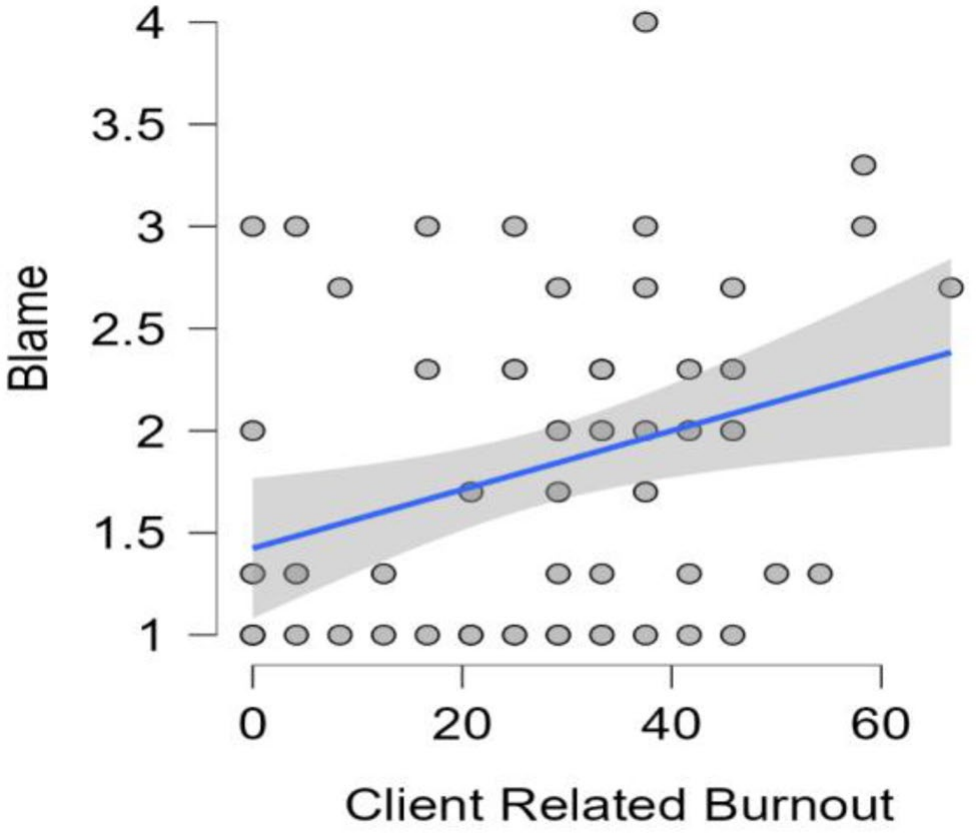
Scatter Plot of the relationship between PBS variant Blame and Client Related Burnout Scores with Linear Regression Line and 95% CI

Three backward multiple linear regression analyses were conducted to assess the predictive relationship of PBS variants on burnout subscales (PB, WB, CB). For PB and WB the final regression models were not significant (*p* > 0.5). However, for CB, the model with *blame* as the predictor was found significant and explained 9.1% of the variance in CB scores *F*(1, 62) = 7.33, *p* = 0.009, with *blame* being a significant positive predictor of CB (*b* = 7.35, *t* = 2.71), *p* = 0.009 (Table 5; Fig. 3).

**Table 5:** Multiple regression analysis predicting client related burnout and HRAS scores from Providers-Based stigma variants

Outcome Variable	Predictor	b	SE (b)	β	t	*p*	95% CI LL UL	Model Fit		
								*R* ^2^	F (df)	*p*
CB ^a^	Intercept	13.87	5.35		2.59	0.012	3.18 24.57			
	*Blame*	7.35	2.71	0.33	2.71	0.009	1.92 12.77			
								0.09	7.33 (1, 63)	0.009
HRAS	Intercept	0.69	0.19		3.58	< 0.001	0.34 1.08			
	*Dangerousness*	0.41	0.10	0.45	4.10	< 0.001	0.21 0.61			
	*Blame*	0.23	0.09	0.29	2.67	0.010	0.06 0.40			
								0.37	19.61 (2, 63)	< 0.001

Note. ^*a*^ *Client-Related Burnout measured by Copenhagen Burnout Inventory*

*CB = Client Related Burnout*,* HRAS = Harm Reduction Acceptability Scale*,* b = unstandardised regression coefficient*,* β = standardised regression coefficient*,* 95% CI LL*,* UL = lower and upper limits of a confidence interval for b*,

### Attitudes towards addiction treatment approaches: analysis of HRAS scores

To investigate attitudes towards addiction treatment approaches a one sample t-test was conducted to investigate whether the HRAS score means differed from neutral midpoint (*M* = 3), with lower scores (*M* < 3) indicating higher acceptance of *harm reduction-based treatments* and higher scores (*M* > 3) indicating higher *acceptance of abstinence-based treatments*.

Results indicated that participants were more likely to accept *harm reduction-based treatments* than *abstinence-based treatments* in this sample *t*(63) = -15.36, *p* <. 001, *Cohen’s d* = 1.9 (Table 6).

**Table 6:** One sample t-test results for HRAS scores and job satisfaction scores

Variable	M (SD)	MD	t (df)	*p*	95% CI	Cohen’s d
					LL, UL	
HRAS	1.84 (0.6)	-1.16	-15.36 (63)	< 0.001	-1.32, -1.01	1.55
Job Satisfaction ^**a**^	4.1 (0.72)	1.11	12.41 (63)	< 0.001	0.93, 1.29	-1.92

Note. ^**a**^ Measured by Job Satisfaction item “How satisfied are you with your current job”

*HRAS = Harm Reduction Acceptability Scale*,* MD* = mean difference, 95% CI LL, UL = lower and upper limits of a confidence interval for MD

### Relationship between PBS and HRAS scores

**Fig. 4:**
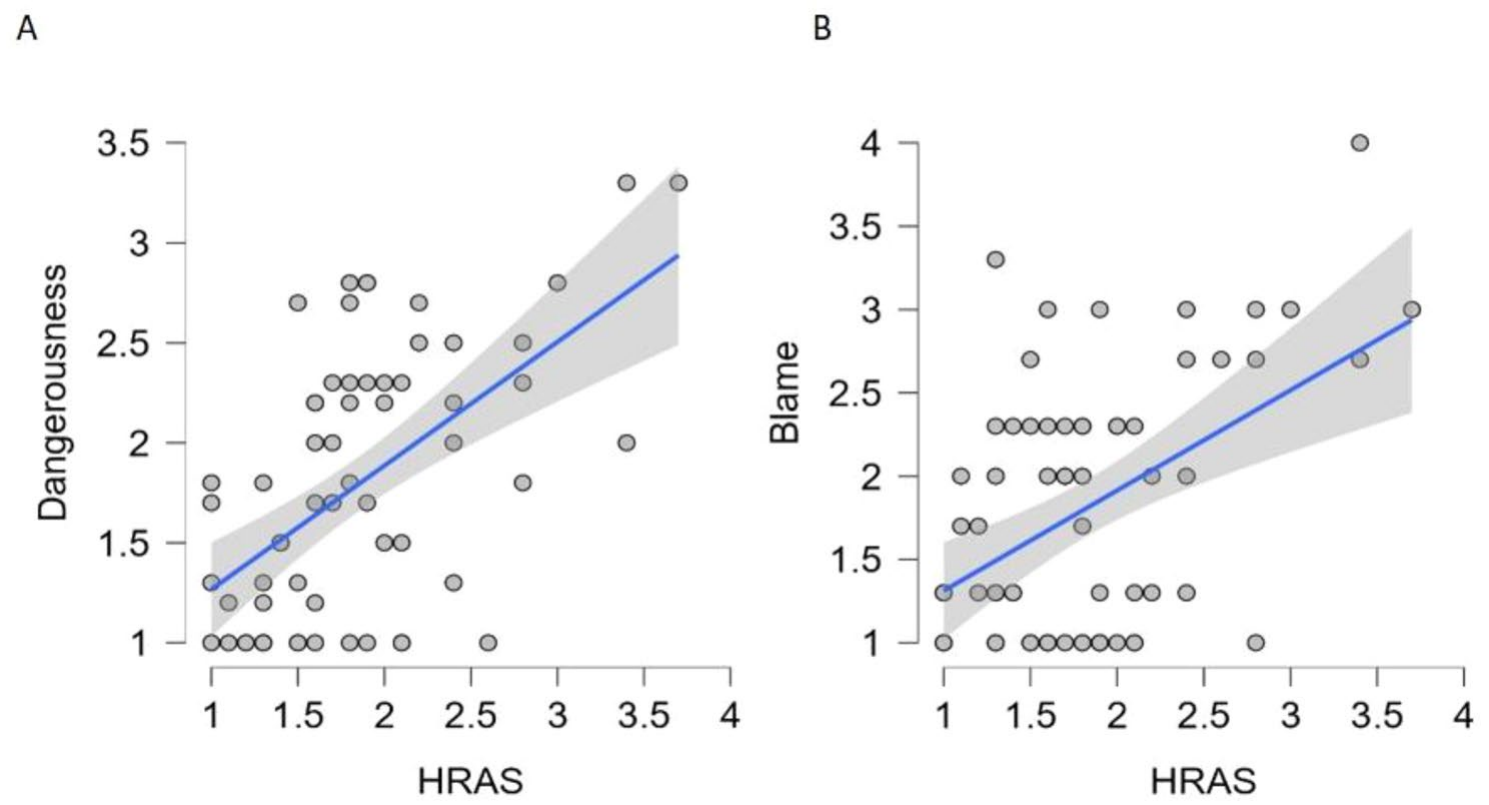
Scatter plots displaying the relationships between (**A**) PBS variant Dangerousness and HRAS score (**B**) PBS variant Blame and HRAS scores, with linear regression lines and 95% confidence intervals

A backward multiple linear regression was conducted to identify the most important predictors of HRAS scores from the four PBS variants. The final model included 2 predictors, *Dangerousness* and *Blame* and explained 37.1% of the variance in HRAS scores *F*(2, 61) = 19.61 *p* <. 001 (Table 5). Results indicated that *Dangerousness* (*b* = 0.41, *t* = 4.10, *p* < 0.001), and *Blame* (*b* = 0.23, *t* = 2.67, *p* = 0.010; Table 5; Fig. 4) were positive predictors of higher HRAS scores i.e. higher acceptance of *abstinence-based treatments*, and lower acceptance of *harm reduction-based treatments.*

### Job satisfaction

A one sample t-test was conducted to determine if participants’ job satisfaction score was significantly different from neutral score (*M* = 3) with results indicating that job satisfaction was high in this sample *t*(63) = 12.41, *p* < 0.001 *Cohen’s d* = 1.5 (Table 6).

### Relationship between PBS and job satisfaction

Spearman’s rho correlation was conducted to examine the relationship between job satisfaction and the four PBS variants, with results showing that only *Fatalism* had a significant negative correlation with job satisfaction (*r* = -0.32, *p* = 0. 011).

### Addiction aetiology

Repeated measures ANOVA was conducted to examine the differences in mean scores on addiction aetiology, between the belief in the *psychosocial model* and the *disease model*. A significant main effect of Addiction Aetiology was found F(1, 63) = 100.01, *p* < 0.001, *η²* = 0.61. Post hoc revealed that participants had significantly higher mean scores on the *psychosocial model* (*M* = 2.88, *SD* = 0.67) compared to the *disease model* (*M* = 1.34, *SD* = 1.06, See Table 7).

**Table 7:** Repeated measures ANOVA for addiction aetiology belief with Post-hoc comparisons (Holm-Bonferroni Correction)

Measure	SS	df	MS	F	*p*	η²	Post Hoc Test			
							MD	95% CI LL UL	t	*p*
Addiction Aetiology^a^	75.81	1, 63	75.81	100.01	< 0.001	0.61				
*Psychosocial Model* vs. *Disease Model* ^a^							-1.54	-1.85 -1.23	-10.00	< 0.001

Note. ^a^Measured by Understanding Substance Use Scale (USUS)

*SS* = sum of squares, *MS* = mean square, *MD* = mean difference, 95% CI LL, UL = lower and upper limits of a confidence interval for *MD*

### Addiction aetiology beliefs and their relationship with attitudes towards addiction treatment approaches

To investigate the relationship between addiction aetiology beliefs and attitudes towards addiction treatment approaches, first Pearson’s correlation was conducted, examining the relationship between scores on the belief in the *psychosocial model*, belief of the *disease model* and HRAS scores.

There was a significant weak, negative correlation between belief in the *psychosocial model* and HRAS scores (*r* = -0.3, *p* <. 05) indicating that higher scores on *psychosocial model* correspond to lower scores on HRAS i.e. higher acceptance of *harm reduction-based treatments*.

The *disease model* had a moderate positive correlation with HRAS scores (*r* = 0.51, *p* < 0.001) indicating that greater scores on the *disease model* corresponded to higher scores on HRAS i.e. higher acceptance of *abstinence-based treatments*.

**Fig. 5:**
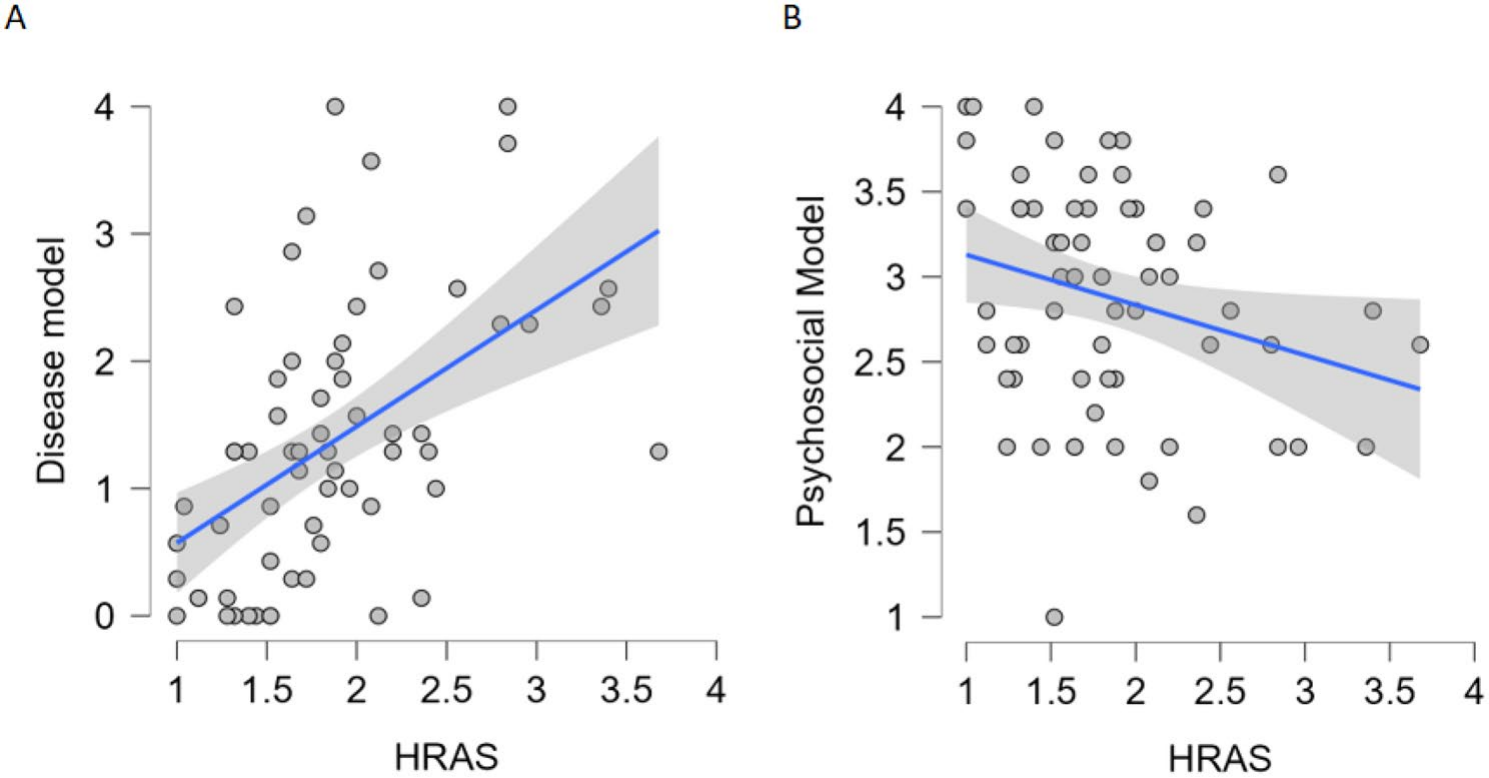
Scatter plots displaying the relationships between (**A**) the belief in the disease model and HRAS scores, (**B**) the belief in the psychosocial model and HRAS scores, with linear regression lines and 95% confidence intervals

Furthermore, a multiple regression analysis was performed to evaluate the predictive effect of addiction aetiology beliefs on HRAS scores. The multiple linear regression showed that *psychosocial model* and *disease model* significantly explained 33% of the variance in HRAS scores *F*(2,61) = 16.19, *p* < 0.001. The belief in *psychosocial model* was a significant negative predictor (*b* = -0.25, t = -2.73, *p* = 0.008) and the belief in the *disease model* of addiction was a significant positive predictor (*b* = 0.30, *t* = 5.09, *p* < 0.001) of HRAS scores (Table 8; Fig. 5). This indicates that the *belief in psychosocial* model predicted higher acceptance of *harm-reduction based-treatment*, whereas the belief in the *disease model* predicted higher acceptance of *abstinence-based treatments*.

**Table 8:** Multiple regression analysis predicting HRAS scores from the disease and psychosocial model belief measures

Outcome Variable	Predictor	b	SE (b)	β	t	*p*	95% CI LL UL	Model Fit		
								*R* ^2^	F (df)	*p*
HRAS	Intercept	2.17	0.28		7.70	< 0.001	1.60 2.74			
	Psychosocial Model^a^	-0.25	0.09	-0.28	-2.73	0.008	-0.44 -0.07			
	Disease Model^b^	0.30	0.06	0.52	5.09	< 0.001	0.18 0.41			
								0.33	16.19 (2, 63)	< 0.001

Note. ^a^Measured by Understanding Substance Use Scale (USUS)

^b^Measured by Understanding Substance Use Scale (USUS)

HRAS = Harm Reduction Acceptability Scale, *b* = unstandardised regression coefficient, *β* = standardised regression coefficient, 95% CI LL, UL = lower and upper limits of a confidence interval for *b*

### Addiction aetiology beliefs and their relationship with PBS

**Fig. 6:**
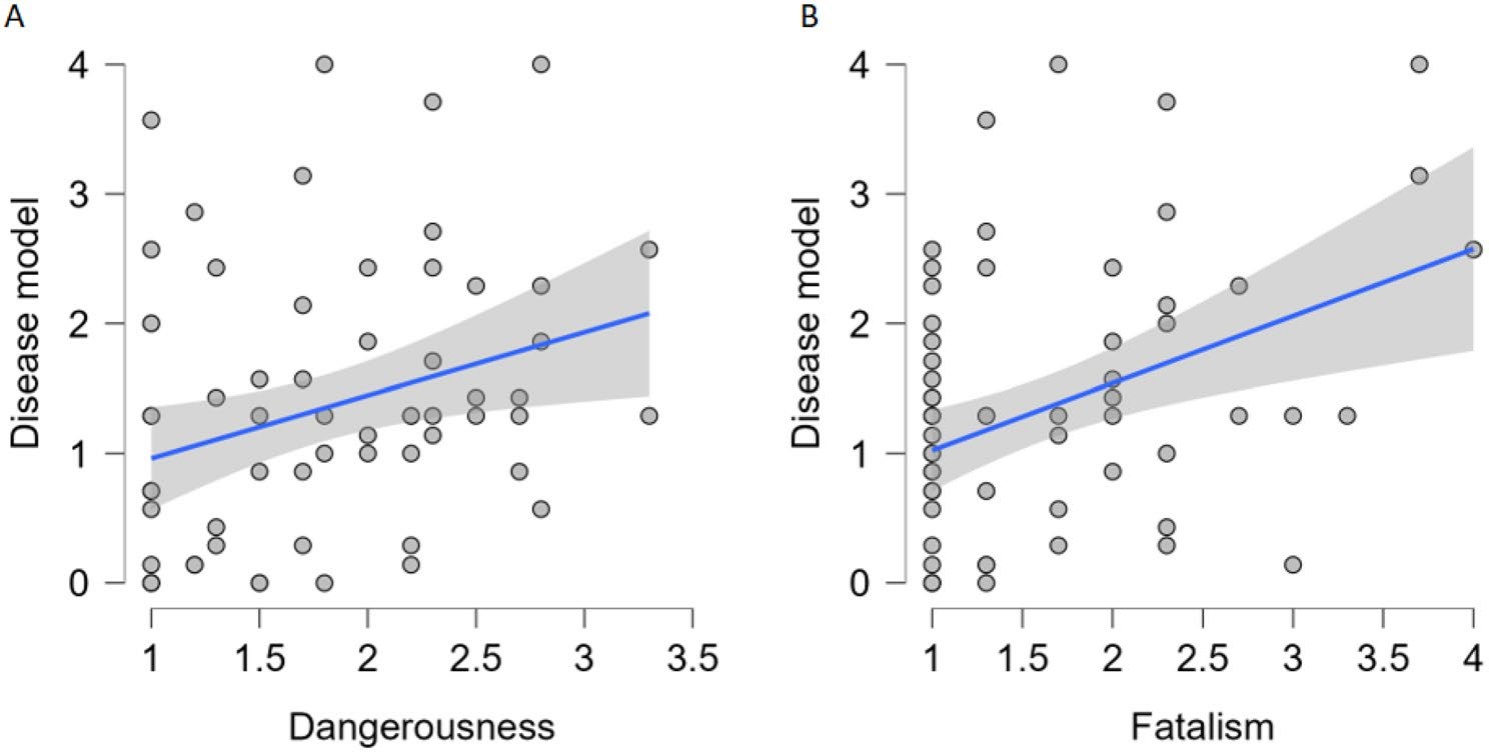
Scatter plots displaying the relationships between (**A**) Dangerousness and the belief in the disease model scores, (**B**) Fatalism and the belief in the disease model scores, with linear regression lines and 95% confidence intervals

Multiple Linear Regression models were performed to evaluate predictive relationship of addiction aetiology on each stigma variant. Only models for *Dangerousness* and *Fatalism* were significant. For *Dangerousness*, the model explained 6% of the variance *F*(2,61) = 3.1, *p* = 0.05, with the *disease model* being a significant positive predictor (*b* = 0.19, *t* = 2.48), *p* = 0.016. For *Fatalism*, the model explained 14% of the variance *F*(2, 61) = 6.24, *p* = 0.003 with the *disease model* being a significant positive predictor (*b* = 0.29, *t* = 3.27), *p* = 0.002 (Table 9; Fig. 6). The *psychosocial model* was not found to significantly predict either PBS variant (*p* > 0.05), (Table 9).

**Table 9:** Multiple regression analysis predicting PBS variants (*Dangerousness*,* Fatalism*) from the scores on beliefs in the *Disease model* and *Psychosocial model*

Outcome Variable	Predictor	b	SE	β	t	*p*	95% CI LL UL	Model Fit		
								R^2^	*F* *(df)*	*p*
Dangerousness	Intercept	1.42	0.37		3.84	< 0.001	0.68 2.15			
	Disease Model^a^	0.19	0.08	0.30	2.48	0.016	0.02 0.34			
	Psychosocial Model^b^	0.04	0.12	0.04	0.33	0.741	-0.20 0.28			
								0.06	3.16 (2,63)	0.049
Fatalism	Intercept	0.74	0.42		1.76	0.083	-0.99 1.59			
	Disease Model	0.29	0.09	0.38	3.27	0.002	0.11 0.46			
	Psychosocial Model	0.17	0.14	0.14	1.23	0.223	-0.11 0.45			
								14.3	6.25 (2,63)	0.003

Note. ^a^Measured by Understanding Substance Use Scale (USUS)

^b^Measured by Understanding Substance Use Scale (USUS)

*b* = unstandardised regression coefficient, *β* = standardised regression coefficient, 95% CI LL, UL = lower and upper limits of a confidence interval for *b*

## Discussion

This cross-sectional survey study investigated provider-based stigma (PBS) and its relation to burnout, job satisfaction, attitudes towards addiction treatment approaches, and addiction aetiology beliefs among Scottish addiction treatment providers. Results indicate that increased levels of PBS have a negative impact on burnout, job satisfaction, and influence providers views regarding addiction treatment approaches. Furthermore, addiction aetiology beliefs among providers were found to influence views on delivery and quality of addiction treatment in Scotland.

The overall levels of PBS were relatively low in our sample, however over 30% of participants did show elevated perceptions of dangerousness and blame towards individuals with SUD. This supports previous work which has shown that treatment providers often see individuals with SUD as dangerous and blameworthy, rather than having perceptions that they are incapable of recovery [21–24]. Desire to keep social distance was low in our sample, which is contrary to previous work [21–24]. This difference in findings could be related to societal and cultural differences between Scotland and the US. To our knowledge, this is the first investigation of the four PBS variants in Scottish addiction services, therefore further research is necessary, especially given the evidence that PBS can negatively affect the quality of addiction services [15, 16, 18, 36].

In line with previous research [18, 37] we found moderately high levels of burnout, especially Personal Burnout (PB) and Work-Related Burnout (WB). This highlights a potential risk to those engaging with Scottish addiction services. Burnout can negatively affect support provided, through impacting staff retention and provider-client relationships [8, 38, 39]. Notably, our findings also indicate that providers who blamed individuals with SUD for their condition were more likely to experience Client-Related Burnout (CB). Research in this area is limited but previous healthcare studies indicated that despite the evidence that SUDs develop from combined biological and social factors [40–42] many providers blame patients for their condition, causing lower empathy towards patients, poor work ethic and quality care [43, 44]. These findings show that stigmatising attitudes could negatively affect addiction treatment providers, by impacting their burnout levels. This emphasises the need for further investigation into PBS, and its relationship with burnout in Scottish addiction services.

Job satisfaction was high in our sample, which could be due to the low levels of CB. Research shows that high job satisfaction often occurs when providers can make meaningful connections with their patients, but experience burnout when encountering administrative and workload-related problems when providing care [45, 46]. Further investigation of the relationship between job satisfaction and burnout is warranted. We investigated the relationship between job satisfaction and PBS and found a correlation between fatalistic views regarding SUD recovery and lower job satisfaction. Research in this area is limited but indicates that treatment providers who are working within recovery-oriented environments have significantly higher job satisfaction [47–49]. This provides a target for treatment improvements, as adapting recovery-oriented programmes in Scottish addiction services could potentially improve job satisfaction and promote recovery optimism.

Harm reduction acceptance was found to be higher in this sample compared to acceptance of abstinence-based treatments. Harm reduction approaches have become increasingly popular in Scotland, with expanding needle exchange programmes, naloxone provisions and the opening of the first safe drug consumption room [3–5, 50] which all have been shown to significantly reduce risk of overdose and drug related harms [51, 52]. Our study investigated the extent to which PBS could influence attitudes towards harm reduction approaches. Higher perceptions of dangerousness and blame were found to predict lower acceptance of harm reduction approaches and heightened the acceptance of abstinence-based treatments. This supports previous research, showing that providers who blamed patients for their condition, and perceived them as dangerous were less likely to accept MAT and Naloxone provisions [22, 23, 53, 54]. This in turn suggests that PBS can affect harm reduction acceptance, revealing a need for policy and education improvements that reduce stigmatising attitudes among Scottish treatment providers, ensuring further expansion and acceptance of harm reduction-based treatments.

We also established providers beliefs in addiction aetiology. The belief in psychosocial model of addiction was more common in this sample compared to the disease model. This aligns with recent evidence that UK-based addiction treatment providers are more likely to endorse psychosocial views compared to the US, where abstinence beliefs are more common [55]. Notably, some participants in our sample still exhibited high belief in the disease model, suggesting a disparity in beliefs between providers. These discrepancies could be problematic as research shows that differential beliefs in addiction aetiology could translate to biased client assessment or favouring certain treatment approaches over others [25–27, 55]. This could lead to clients receiving inconstant treatments, or contradictory explanations for their condition, affecting their treatment satisfaction and engagement [27, 55].

Our data also showed that the beliefs in addiction aetiology were significant predictors of accepting different treatment approaches, with the disease model significantly predicting higher acceptance of abstinence-based treatments, and psychosocial model predicting higher harm reduction acceptance. Belief in the disease model was also a predictor for increase in fatalistic views regarding recovery, and perceptions that individuals with SUD are dangerous. This challenges previous work which found the belief in the disease model was positively associated with harm reduction endorsement and increased recovery optimism [22, 23, 54]. Our results also support previous findings showing belief in the disease model was associated with lower harm reduction acceptance [27, 55] as well as increased perceptions of dangerousness and recovery fatalism [27, 30, 55]. Evidently, research is limited and demonstrates inconsistencies, with our data contributing to this. However, overall, it reinforces the idea that providers beliefs could translate into different, biased practices and influence quality and delivery of treatments. There is a need for further investigation, given the evidence that addiction aetiology beliefs could affect acceptance of evidence-based treatments and negatively affect therapeutic alliances by heightening PBS.

### Limitations

The study has limitations that need to be considered. Firstly, the cross-sectional design of this study limits our ability to assume causality. Longitudinal data would be necessary to clarify our results. Secondly, the sample size and response rate could have been improved, limiting the possibility of non-response bias in our results however, the targeted group is difficult to reach which is evidenced by lack of research involving Scottish addiction treatment providers. Furthermore, the low response rate has been noted in previous studies recruiting this population [56–58]. Future research in this area should investigate the causes of this ongoing recruitment bias.

The study also adapted an existing PBS measure, originally developed for providers working with individuals with opioid use disorder to apply to individuals with any SUD. Whilst the original PBS measure is showing promising validity [21–24], it is a new measure and adapting the wording to all SUDs could have impacted its accuracy, given that certain constructs tailored to opioid use may not apply to all SUDs. However, the measure benefits from multidimensional investigation of PBS lacking in previous research. Further research is needed to validate this adaptation and examine possible sensitivity of the measure to different types of SUDs to strengthen its robustness and generalisability. Lastly, our overall low scores on this measure could have limited its sensitivity to detect associations, which should be considered when interpreting the findings.

### Conclusion and future directions

Our findings provide data that could support improvements in approaches to addiction treatment in Scotland. Firstly, we have shown that PBS still exists within Scottish addiction workforce, especially the perception that individuals with SUD are dangerous and blameworthy. Further results indicate that certain variants of PBS are negatively related to burnout, job satisfaction, and lower acceptance of harm reduction approaches. As all these aspects can negatively impact the accessibility, quality, and delivery of addiction services, this highlights the need for efforts to alleviate these stigmatising attitudes. At the individual level, stigma reduction workshops could limit treatment bias, and burnout, fostering better provider-client relationships. At the organisation and policy level, improving staff mentoring and ensuring achievable workloads could also minimise the occurring PBS and in turn enhance the success of Scottish addiction services.

Furthermore, it is important for policy makers responsible for addiction workforce development in Scotland to be aware of treatment providers views regarding addiction aetiology. Discrepancies exist, and our results show that certain beliefs (e.g. belief in the disease model) could translate to favouring certain treatment approaches (e.g. abstinence-based) and heighten the level of PBS, which as previously noted can have further negative impact on treatment delivery and quality. Future research should explore how different providers views can affect client engagement and provider-client relationships in Scottish addiction services. Overall, this is one of the first studies to explore PBS and addiction aetiology beliefs and their impact on variables related to quality and delivery of addiction treatments in Scotland. Our results highlight several areas of improvement but given the novelty of this research there is an urgent need for further exploration and development of these findings. Ongoing work is investigating this area in more detail by conducting semi-structured interviews with stakeholders in the field.

## Data Availability

The datasets generated and analysed during the current study are not publicly available due to the sensitive nature of the datasets but are available from the corresponding author on reasonable request.

## Abbreviations

CB: Client Related Burnout
CBI: Copenhagen Burnout Inventory
HRAS: Harm Reduction Acceptability Scale
MAT: Medication Assisted Treatment
PB: Personal Burnout
PBS: Provider Based Stigma
SUD: Substance Use Disorder
WB: Work Related Burnout
